# Advances in Understanding the Causes, Molecular Mechanism, and Perspectives of Russeting on Tree Fruit

**DOI:** 10.3389/fpls.2022.834109

**Published:** 2022-02-28

**Authors:** Shenghui Jiang, Min Chen, Ziqi Wang, Yanxue Ren, Bin Wang, Jun Zhu, Yugang Zhang

**Affiliations:** ^1^Engineering Laboratory of Genetic Improvement of Horticultural Crops of Shandong Province, College of Horticulture, Qingdao Agricultural University, Qingdao, China; ^2^Yantai Institute of Coastal Zone Research, Chinese Academy of Sciences, Yantai, China

**Keywords:** fruit russeting, causes, histomorphology, composition, genetics, regulation

## Abstract

The external quality of fruit is one of its most important qualities; good external quality attracts consumers easily and increases the value of fruit. Fruit russeting is one of the factors that influences the external quality of fruit and has been studied in most horticultural plants. However, the molecular mechanism of russeting has never been discussed so far. In this review, we summarize the research progress on fruit russeting, including causes, microscopic histomorphology, composition, genetics, and regulation and made a series of elaboration on the current research on fruit russeting. This study aims to provide insights into the mechanisms underlying fruit russeting. It also puts forward ideas for research on fruit russeting, which may provide a reference for future research.

## Introduction

**FIGURE 1:**
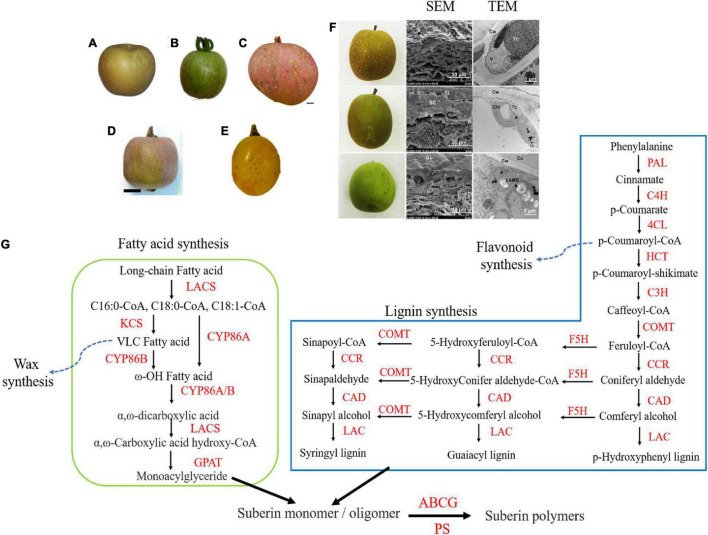
The russet symptoms of different fruits and the proposed model of russeting in tree fruit. **(A–E)** The russet symptom of “Rugiada” apple, SlDCR-RNAi tomato, “Apple” mango, kiwifruit, and “Sunshine Muscat” grape (derived from Lashbrooke et al., 2016; Athoo et al., 2020; Huang et al., 2020; Falginella et al., 2021; Macnee et al., 2021, respectively). **(F)** The symptoms and microscopic histomorphology of “Zaoshengxinshui” (upper), “Cuiguan” (middle), and “Cuiyu” (lower, derived from Wang et al., 2021). **(G)** A proposed model of russeting in tree fruit. The lignin synthetic and fatty acid synthetic pathway provide precursors for suberin synthesis with other compounds during russeting. The compounds of lignin pathway including phenylalanine, cinnamate, ferulate, and coniteryl aldehyde and the compounds of fatty acid pathway including long-chain fatty acid and ω-hydroxyacid usually increase in russet fruit. The wax synthetic and flavonoid synthetic pathway were suppressed because of the accumulation of long-chain FA and lignin. PAL, Phenylalanine ammonia-lyase; C4H, cinnamate-4-hydroxylase; 4CL, 4-coumaric acid: CoA ligase; HCT, lignin synthase; COMT, caffeic acid 3-O-methyltransferase; C3H, coumaryl coenzyme A3-hydroxylase; F5H, flavonoid 5-hydroxylase; CCR, cinnamoyl-CoA reductase; CAD, cinnamyl alcohol dehydrogenase; LAC, laccase; LACS, long-chain acyl-CoA synthetase; KCS, β-ketoacyl-CoA synthase; GPAT, glycerol-3-phosphate acyltransferase; ABCG, ATP-binding cassette subfamily G; PS, polyester synthase.

Russeting is an important physiological disorder that can compromise the external quality of fruit and reduce its commercial value. It is commonly found on pear and apple (Figures 1A,F). Because consumers prefer smooth textured and colorful fruits, fruits with russeting are non-desirable. Russeting also increases loss of moisture after post-harvest, thereby affecting shelf life, storage, and transport. Methods to prevent russeting include bagging, using phytohormones (e.g., BA and GA), and other measures (Alston and Watkins, 1973; Heng et al., 2016; Wang et al., 2021). Research on russeting mainly involved studying the suberin, cutin layer, and lignin biosynthetic pathway (Lashbrooke et al., 2016; Wang et al., 2020; Ma et al., 2021; Shi et al., 2021). Although a review of the molecular research of russet/semi-russet of sand pear was reported previously (Wang et al., 2016), the molecular research of russeting in tree fruit has not been discussed till now. In this article, we summarize previous studies of causes, histomorphology, composition, genetics, and regulation of fruit russeting in order to provide insights for further research on the underlying mechanism of russeting in tree fruit.

## Causes of Fruit Russeting

Environmental and cultural factors influence russeting. For example, light intensity influences the incidence of fruit russeting by affecting the levels of endogenous gibberellins. This also explains the significant differences in russet plant development observed with altitude changes (Eccher, 1986; Noè and Eccher, 1996). Water could also induce microscopic cracks in the cuticle and increase russeting of fruit surface (Knoche et al., 2011; Shi et al., 2021). The susceptibility of fruit to russeting is also dependent on the variety and clone of fruit (Eccher, 1978; Maas, 2015). The fruitlet thinning chemicals and plant protection could also cause russeting of fruit (Table 1; Wertheim, 1986; Teviotdale et al., 1997). In addition, as a biotic cause, pathogens could also cause the development of russeting (Table 1; Bertschinger et al., 1999; Gildemacher et al., 2004). Taken together, russeting may be influenced by both biotic and abiotic causes.

**TABLE 1:** Causes of russeting and countermeasures.

Causes of russeting	Recommended countermeasures
Fungal species: *Aureobasidium pullulans* or *Rhodoturula glutinis* (Matteson-Heidenreich et al., 1997; Gildemacher et al., 2004)	Applying fungicides
Environmental factors: light intensity (Eccher, 1986; Noè and Eccher, 1996); humidity (Shi et al., 2021)	Bagging
Varieties and rootstocks (Maas, 2015)	Selecting the anti-fruit russeting varieties and rootstocks
Copper spray (Teviotdale et al., 1997)	Using lower doses or less applications of copper spray
Fruitlet thinning chemicals (NAAm or carbaryl; Wertheim, 1986)	Mixed with anti-fruit russeting agent GA_4+7_ + BA

## Histomorphology of Fruit Russeting

The surface of russet fruits is always brown, rough, and cracked. Scanning electron microscopy (SEM) shows that the cuticle dramatically reduces in russet fruits leading to such a phenotype. In sand pear, fruit russeting is a unique feature mainly due to the accumulation of suberin lamellae in the peel. The russet skin of sand pear was stratum corneum cracked into pieces that extended to reach the epidermal cells. Meanwhile, the peels were covered by layers of dead cells which is why the outer layer exhibits a loose lamellar structure. Cracks of cuticle were also found in the peel of semi-russet “Cuiguan” pear filled with cork tissues. However, the cuticle was smooth without any cracks in the peel of a non-russet fruit, which consisted of a thick waxy layer (Figure 1F; Wang et al., 2020; Shi et al., 2021). Transmission electron microscopy (TEM) analysis revealed substantial tylosis in the epidermal cells of russet skin of sand pear (Wang et al., 2020). The cuticles of the “Conference” pear peels were cracked and encrusted with suberin (Khanal et al., 2013). A special apple cultivar “Egremont Russet” was popular with the Victorians with slightly tough, brownish-green skin covered by golden russet. No-russeting apple skins generally exhibited neatly arranged cells, a uniform wax layer, and a tight stratum corneum layer with a few gaps (Knoche and Grimm, 2008; Legay et al., 2015). The waxy layer of the epidermis of russet skin was warped with microcracks and the microcracks became bigger and deeper with the “Golden Delicious” fruit development. However, the skin of apple fruits (bagged from May to September) was smooth without microcracks (Yuan et al., 2019). Meanwhile, the cuticle of a mutational sports “Rugiada” apple of “Golden Delicious” showed microcracking between epidermal cells, with the suberin and lignin deposition forming periderm (Figure 1A; Lashbrooke et al., 2016; Falginella et al., 2021). In “Cuiguan” pear, the semi-russet pear skins had a defective cuticle layer, russet-deposited layer, and periderm layer compared with those of the bagged (non-russet) pear skins. Semi-russet pear fruit skins contained more lipid components in place of lignin than those of bagged fruit skins (Zhang et al., 2021). Moreover, the browning spot in “Huangguan” pear exhibited a very similar pattern to that of russet pears: the degree of lignification of the exocarp cells of the browning spot parts was significantly higher compared with that of the normal parts and the cuticular layer was much thinner with dead cells and dense exocarp cells (Wang et al., 2021).

Russeting patterns have also been reported in other fruits. For example, when the expression of *DEFECTIVE IN CUTICULAR RIDGES (DCR)* gene, which encodes an acyltransferase of BAHD (BEAT, AHCTs, HCBT, and DAT) family, was suppressed in tomato, the skin of tomato showed cracking and browning, potentially indicative of suberin formation. SEM indicated that the cells of fruit surface had microscopic cracks and large fissures, while TEM showed that lipid inclusion bodies were formed in the fruit epidermal cells (Figure 1B; Lashbrooke et al., 2016). The russeting of mango fruit began at the lenticels; lenticels ruptured and then developed into several stellate cracks that were filled with periderm. These cracks propagated and development progressed eventually forming crack networks that extended over the entire fruit (Figure 1C; Athoo et al., 2020, 2021). The russet skins in the backcross population of kiwifruit were covered with suberized and lignified cells, and the suberin was found under the epidermal layer (Figure 1D; Macnee et al., 2021). Though Huang et al. (2020) reported russeting in grape skin, they did not perform a histomorphology analysis (Figure 1E). We speculate that russet grape skin would also appear rough and cracked under the electron microscopy.

## Composition of Fruit Russeting

The omics research provides wide data for different phenotypes from different aspects. Especially, metabolomics is an effective means to explore the composition of fruit russeting. For example, on chemical characterization of surface of *SlDCR*-RNAi tomato fruit, cutin monomers were found to be reduced significantly, especially the C16-9/10,16-dihydroxyhexadecanoic acid; DHFA (C16-9/10,16-DHFA), a major cutin monomer in tomato (Mintz-Oron et al., 2008; Pollard et al., 2008; Lashbrooke et al., 2016). At the same time, several constituents of suberin, including the terminally hydroxylated fatty acids (FAs; C16-ω-HFA), dicarboxylic FAs (C16:0 dicarboxylic FA) and phenolic, ferulic acid as well as the non-polymerized wax component C18-C24 ferulic esters increased significantly in *SlDCR*-RNAi fruit surface (Pollard et al., 2008; Schreiber, 2010; Lashbrooke et al., 2016). The mid-chain hydroxylated FA (C16-9/10,16-DHFA), terminal hydroxylated FA (C16-ω-HFA), and epoxy FA (C18:1-9,10-epoxy-19-ω-HFA) were drastically reduced, while saturated C22:0 FA, C20- and C22-ω-HFAs increased massively in “Rugiada” apple skin. Meanwhile, metabolites including phenolics, ferulic acid, benzoic acid, and cinnamic acid also increased, indicating suberin formation and their contribution to russeting in apple (Franke et al., 2005; Lashbrooke et al., 2016; Busatto et al., 2019). In pear, the cutin and suberin were considered the main components of russet fruit skins and metabolomic results indicated that cutin contents were reduced and suberin contents were increased resulting in the russet “Cuiguan” pear. The cutin monomers C16 ω-hydroxyacids were mainly reduced, while several suberin monomers, including ferulate, alcohols, FAs, α, ω-dicarboxylic acids, and ω-hydroxyacids, significantly accumulated in the russet skins (Zhang et al., 2021).

In addition, suberized skin tissue of russet apple contains more lupane derivatives, a specific triterpene, and lower ursane and oleanane triterpene types (Boyer and Liu, 2004; Andre et al., 2013; Falginella et al., 2021). Triterpene caffeates have been detected in suberized tissues, such as russet apple skin, but not in waxy, non-suberized apple skin (Schreiber, 2010; Brendolise et al., 2011; Andre et al., 2013). For example, two apple sports, non-russet “Smoothee” and fullyrusset “Rugiada,” were selected from “Golden Delicious.” The contents of ursolic and oleanolic acids in “Rugiada” skin were significantly lower than that in “Golden Delicious” and “Smoothee” from 40 DAFB to 159 DAFB. Conversely, the contents of betulinic acid and betulinic acid-3- *trans*-caffeate in “Rugiada” skin were the highest of the three cultivars (Falginella et al., 2021).

## Genetics and Regulation of Fruit Russeting

In Japanese pears (*P. pyrifolia*), a model of two dominant genes controlling russeting was first reported: the *R* site was responsible for the development of fruit russeting, while the *I* site suppressed suberin formation (Kikuchi, 1930). In apple, the *Ru* gene was considered as the first gene that determined fully russeting alone; however, the non-fully russeting phenotype was found to be controlled by multiple factors by analyzing the phenotypes of offspring that were hybrid among fully, non-fully, and less russeting varieties (Alston and Watkins, 1973). A genetic mapping of segregating progeny of “Renetta Grigia di Torriana” was constructed; the genetic mapping shows that a major genetic determinant of russeting is on linkage group (LG) 12 (Falginella et al., 2015). Meanwhile, researchers found seven major quantitative trait loci (QTL) intervals associated with cuticle in a full-sib population that were generated between “Golden Delicious” and “Braeburn”; which were found to be located on Chromosome (Chr) 2 and Chr 5 *via* association analysis (Lashbrooke et al., 2015). A specific-locus amplified fragment (SALF) genetic map was constructed using “Miyazaki Spur,” “Sakata Tsugaru,” and their progeny, and nine QTLs related to russeting were obtained, which were located on Chr 3, 9, 11, and 15. From those QTLs, 127 genes are annotated (Zhang et al., 2019). In kiwifruit, epidermal skin was found to be a recessive trait on analyzing the phenotype of population crossed between epidermal and peridermal skinned kiwifruit. QTL analysis of this population showed that russeting loci were located on Chr 3, 19, and 23 (Macnee et al., 2021). In a recent study, BSA-seq (RNA-seq-based bulked segregant analysis) was performed, in which linkage analysis found that the *PpRus* locus is located on Chr 8 (Ma et al., 2021). As these results indicate that hybrid groups are appropriate materials to study russeting in fruit tree, they may provide more unique phenotypes for further study.

In order to explore the regulatory genes involved in russeting, the transcriptome strategy is adopted. The differentially expressed genes (DEGs) are mainly enriched in phenylpropanoid biosynthesis, lignin, cutin, suberin and wax biosynthesis, as well as fatty acid biosynthetic and triterpene biosynthetic pathways. For example, seven phenylpropanoid biosynthetic genes (including *4CL*, *CSE*, *COMT*, *HCT*, and *CcoAOMT* members) and 12 genes (including *FAR*, *CYP86A/B*, *GPAT*, and *ASFT* family members) required for suberin aliphatic compound biosynthesis were upregulated and were consistent with the content of suberin monomers in the skins of russet “Cuiguan” pear, suggesting that their expressions contributed to suberin accumulation. Other genes including *KCS*, *ABCG*, *PRX*, *GDSL*, and *LTP* are also involved in cutin and suberin pathway. The downregulation of cutin biosynthetic genes along with the upregulation of suberin biosynthetic genes led to “Cuiguan” russeting (Zhang et al., 2021). In the “Huangguan” pear with browning spot, the related genes, *4CL2*, *CAD1*, *CYP84A1*, *4CL1*, *CYP98A2*, and *COMT1*, involved in lignin biosynthesis, were upregulated; however, two genes, *CAD6* and *CCR1*, were downregulated. The differential expressions of these genes led to the upregulation of metabolites of phenylpropanoid biosynthetic pathway. Meanwhile, genes including the *CYP704C1*, *CYP94A1*, *HTH*, *HHT*, *WSD1*, and *FAR3* genes as well as 10 *KCS* family genes involved in wax biosynthesis were downregulated, suggesting that the decrease in wax may be caused by browning spot (Wang et al., 2021). In grapes, on analyzing the transcriptional result of grape varieties with different degrees of fruit russeting, the *PAL* gene was found to be positively correlated with different degree of fruit russeting. The PAL enzyme activity was also lowest in no-russet grapes, indicating that both lower PAL enzyme activity and gene expression contribute to reducing grape russeting (Xu et al., 2019). Metabolomic and transcriptomic association analysis between russet and no-russet grapes indicates that phenylalanine biosynthesis pathway is closely associated with fruit russeting, and the up-regulated expression of genes associated with lignin and quercetin synthesis promotes russeting (Huang et al., 2020).

In addition, the increased expression of *CCoAOMT* in the skin of “Xiusu” led to the accumulation of lignin content, which was one of the important reasons for the russeting formation of “Xiusu” (Li et al., 2012). Further research shows that the lignin biosynthetic genes, including *PAL*, *CCR*, *CAD*, and *POD/PRX*, were up-regulated in “Xiusu,” indicating that these genes were involved in the russeting of “Xiusu” (Heng et al., 2014). Based on BSA-seq of the offspring from “Qingxiang” and “Cuiguan” F1 group, *CCR*, *CAD*, and *POD* genes involved in lignin biosynthesis were found to be candidate genes that responded to the formation of russet pericarps in sand pear (Wang et al., 2014). After pear fruit bagging, the expression and enzyme activity of PAL, 4CL, C4H, CAD, and POD, which are involved in lignin biosynthesis, were inhibited. In addition, fruit russeting was also inhibited (Shi et al., 2019). In apple, the AP2 transcription factor MdSHN3 could positively regulate the biosynthesis of the apple cuticle and inhibit the formation of apple fruit russeting (Lashbrooke et al., 2015). A MYB family transcription factor MdMYB93 was identified through transcriptomic studies of russet and no-russet apples; the overexpression of *MdMYB93* could accumulate a large amount of lignin monomers and precursors in tobacco leaves, suggesting that this gene is able to positively regulate russeting of apple fruit (Legay et al., 2016). Two MYB transcription factors, MYB9 and MYB107, are homologous with MYB93, and can bind to the *4CL5* and *HHT1* promoters of suberin biosynthetic genes to participate in the deposition of suberin in seed skin and peel, and may positively control russeting (Lashbrooke et al., 2016; Gou et al., 2017). *MdPAL* also plays an important role in fruit russeting (Busatto et al., 2019; Yuan et al., 2019). In “Golden Delicious” apple, four lignin biosynthetic genes were associated with russet formation as found *via* a correlation analysis between transcriptomics and proteomics of bagging and non-bagging fruits. A transcription factor MdLIM11 was able to bind to the CCACTTGAGTAC site of *PAL* promoter to inhibit the expression of the *PAL* gene, thereby inhibiting lignin biosynthesis and affecting russeting (Yuan et al., 2019). The oxidosqualene cyclase (*OSC*) genes are associated with lupane-type triterpene concentrations; especially *MdOSC5* gene was highly expressed in “Rugiada,” indicating that it plays an essential role in suberin-associated triterpene synthesis. Further analyses showed that the expression of *MdOSC5* was regulated by MYB52 and MYB66, indicating that MYB52 and MYB66 potentially activate lupine-type triterpene biosynthesis in russet apple (Falginella et al., 2021). In pear, transient expression of *PbHHT1* gene in young green non-russet fruits led to a lenticel suberization genotype with higher content of ω-feruloyloxypalmitic acid (Shi et al., 2021). Using BSA-seq, a MYB transcription factor MYB36 was identified as a regulator that was involved in lignin accumulation and russet coloration in pear (Ma et al., 2021). In addition to the above genes, *ABCGs* were also involved in suberin formation and fruit russeting (Falginella et al., 2015; Hou et al., 2018; Shi et al., 2021), but the functions of this family remain to be determined in fruit tree. Put together, these results indicate that fruit russeting may be associated with transcriptional regulation, FA synthesis, lignin/phenylpropanoid biosynthesis, extracellular polymerization, and transport.

## Conclusion and Future Perspective

Fruit russeting is complex and mainly involves accumulation of suberin in the fruit peels. This phenotype adversely affects the external quality of fruit and reduce its commercial value. Measures to prevent fruit russeting are always followed during fruit production including bagging, applying fungicides, selecting suitable varieties or rootstocks, and applying phytohormones (Table 1). Subsequently, modern technology, including but not limited to fruit grading or sorting, should also be applied when russeting is inevitable (Klemm et al., 2016; Schüsseler et al., 2019). However, further studies need to be performed to understand the mechanism of russeting so that russeting can be prevented early during fruit production.

From this review, we have known about two important pathways, lignin, and fatty acid metabolism, which provide various precursors for suberin synthesis with other compounds during russeting (Figure 1G). This information provides us three strategies to prevent the formation of fruit russeting that may also aid future studies: (i) inhibiting the synthesis of lignin and FAs so that they cannot supply precursors for suberin biosynthesis; (ii) inhibiting the key enzymes involved in the polymerization and transport processes, so that polymerization reaction and transport process do not occur during russeting; and (iii) as lignin synthesis and pigment synthesis share the same pathway, approaches to accumulate more pigments instead of lignin can help stop russeting. Thus, it is particularly important to screen and identify key genes involved in russeting. Besides, molecular markers associated with russeting or no-russeting need to be developed for molecular marker-assisted breeding in fruit tree.

## Author Contributions

SJ and YZ initiated and designed the project. SJ and MC wrote the manuscript. SJ, MC, JZ, and YZ revised the manuscript. ZW, YR, and BW provided assistance to writing this manuscript. All authors approved it for publication.

## Conflict of Interest

The authors declare that the research was conducted in the absence of any commercial or financial relationships that could be construed as a potential conflict of interest.

## Publisher’s Note

All claims expressed in this article are solely those of the authors and do not necessarily represent those of their affiliated organizations, or those of the publisher, the editors and the reviewers. Any product that may be evaluated in this article, or claim that may be made by its manufacturer, is not guaranteed or endorsed by the publisher.
